# Proton and phosphorus magnetic resonance spectroscopy of the healthy human breast at 7 T

**DOI:** 10.1002/nbm.3684

**Published:** 2016-12-28

**Authors:** Wybe J.M. van der Kemp, Bertine L. Stehouwer, Vincent O. Boer, Peter R. Luijten, Dennis W.J. Klomp, Jannie P. Wijnen

**Affiliations:** ^1^ Department of RadiologyUniversity Medical Center UtrechtUtrechtthe Netherlands

**Keywords:** ^1^H, ^31^P, 7 T, breast, MRSI, total choline, transverse relaxation time

## Abstract

*In vivo* water‐ and fat‐suppressed ^1^H magnetic resonance spectroscopy (MRS) and ^31^P magnetic resonance adiabatic multi‐echo spectroscopic imaging were performed at 7 T in duplicate in healthy fibroglandular breast tissue of a group of eight volunteers. The transverse relaxation times of ^31^P metabolites were determined, and the reproducibility of ^1^H and ^31^P MRS was investigated. The transverse relaxation times for phosphoethanolamine (PE) and phosphocholine (PC) were fitted bi‐exponentially, with an added short *T*_2_ component of 20 ms for adenosine monophosphate, resulting in values of 199 ± 8 and 239 ± 14 ms, respectively. The transverse relaxation time for glycerophosphocholine (GPC) was also fitted bi‐exponentially, with an added short *T*_2_ component of 20 ms for glycerophosphatidylethanolamine, which resonates at a similar frequency, resulting in a value of 177 ± 6 ms. Transverse relaxation times for inorganic phosphate, γ‐ATP and glycerophosphatidylcholine mobile phospholipid were fitted mono‐exponentially, resulting in values of 180 ± 4, 19 ± 3 and 20 ± 4 ms, respectively. Coefficients of variation for the duplicate determinations of ^1^H total choline (tChol) and the ^31^P metabolites were calculated for the group of volunteers. The reproducibility of inorganic phosphate, the sum of phosphomonoesters and the sum of phosphodiesters with ^31^P MRS imaging was superior to the reproducibility of ^1^H MRS for tChol. ^1^H and ^31^P data were combined to calculate estimates of the absolute concentrations of PC, GPC and PE in healthy fibroglandular tissue, resulting in upper limits of 0.1, 0.1 and 0.2 mmol/kg of tissue, respectively.

**Abbreviations**
AFP: adiabatic full passage
AHP: adiabatic half passage
AMESING: adiabatic multi‐echo spectroscopic imaging
AMP: adenosine monophosphate
ATP: adenosine triphosphate
FID: free induction decay
FOCI: frequency offset corrected inversion
GOIA: gradient‐modulated offset independent adiabaticity
GPC: glycerophosphocholine
GPE: glycerophosphoethanolamine
GPtC: (diacyl‐)glycerophosphatidylcholine
GPtE: (diacyl‐)glycerophosphatidylethanolamine
MPL: membrane phospholipid
MRS: magnetic resonance spectroscopy
MRSI: magnetic resonance spectroscopic imaging
NMP: nucleoside monophosphate
NTP: nucleoside triphosphate
PC: phosphocholine
PCr: phosphocreatine
PDE: phosphodiester
PE: phosphoethanolamine
Pi: inorganic phosphate
PME: phosphomonoester
RF: radiofrequency
SAR: specific absorption rate
SNR: signal‐to‐noise ratio
tChol: ‘total choline’ (also including ethanolamine)
VAPOR: variable power and optimized relaxation delays

## 1 INTRODUCTION

Elevated concentrations of the phosphomonoesters (PMEs) phosphocholine (PC) and phosphoethanolamine (PE), which are reaction intermediaries in membrane phospholipid synthesis, are a metabolic hallmark of cancer.^1^, ^2^, ^3^, ^4^ Intermediaries of phospholipid breakdown are the phosphodiesters (PDE) glycerophosphocholine (GPC) and glycerophosphoethanolamine (GPE).

Cell membrane metabolism can be monitored *in vivo* by proton (^1^H) and phosphorus (^31^P) magnetic resonance spectroscopy (MRS), which may be used clinically for cancer diagnosis and treatment monitoring.^5^ Unfortunately, the spectral resolution of *in vivo* ^1^H spectroscopy is not sufficient to show the individual PME and PDE resonances. These resonances are within the small chemical shift range of 3.2 and 3.3 ppm, and overlap with other metabolites. Particularly in organs that have tissue compartments with substantially different susceptibility, such as lipids and glandular tissue in breast, even higher field strengths will not sufficiently improve the spectral resolution. Moreover, fluctuations caused by breathing, heartbeat and subject motion complicate *in vivo* shimming of the *B*_0_ field, resulting in further compromises in spectral resolution. Nevertheless, a total choline (tChol) signal can be observed by *in vivo* ^1^H MRS in breast tumors and also in healthy fibroglandular breast tissue. The tChol signal comprises choline, PC, PE, GPC and GPE, but may potentially also be contaminated by signals from β‐glucose, taurine and myo‐inositol as a result of poor spectral resolution in the breast at 7 T. It should be noted that the term ‘total choline’ also includes the PEs.

The application of ^1^H spectroscopy in breast cancer diagnostics and treatment response has been the subject of several reviews,^6^, ^7^, ^8^, ^9^, ^10^, ^11^, ^12^ in which extensive entries to the literature can be found. *In vivo* tChol ^1^H MRS in the human breast is challenging because of the high water and (potentially) high fat signals, which are several orders of magnitude larger than the tChol signal. Appropriate placement of the MRS voxel, excluding as much fatty tissue as possible, is therefore important. In addition, robust water and fat suppression is required,^12^ for which we have chosen a VAPOR (variable power and optimized relaxation delays)^13^ scheme (water suppression) prior to the sequence and two chemical shift‐selective radiofrequency (RF) pulses surrounded by crusher gradients applied within the TE of the MRS sequence (MEGA^14^ scheme fat suppression). At high magnetic field, the most accurate localization technique available so far is a single‐voxel semi‐LASER sequence,^15^ as it uses adiabatic high‐bandwidth RF pulses that have small chemical shift displacement artifacts and slice profiles with sharp transitions. In the case of MR coils and systems with limited *B*_1_, the use of a semi‐LASER with FOCI (frequency offset corrected inversion) or GOIA (gradient‐modulated offset independent adiabaticity) pulses could provide a better alternative to minimize the chemical shift artifact, although the use of these pulses leads to longer TEs and higher specific absorption rate (SAR). In order to effectively incorporate single‐shot frequency alignments to mitigate field fluctuations during the scan time, metabolite cycling can be applied,^16^, ^17^ in which the large signal of water can be removed by subtraction, but used for accurate frequency and phase alignments. When residual water or lipid signals remain visible, Bolan et al^18^ have demonstrated the use of TE averaging to remove potential artifacts originating from side bands caused by gradient‐induced vibrations. Finally, to ensure that cardiac‐induced rapid field fluctuations remain outside of the TE of the sequence, cardiac triggering can be applied as demonstrated by Andreychenko et al.^19^ All of these techniques were incorporated in one scan sequence to enable robust ^1^H MRS in the breast.

Despite the technical advances in robust ^1^H MRS, *in vivo* ^1^H tChol MRS has the severe limitation that it only reflects tChol and not the individual PMEs and PDEs. Although less sensitive than ^1^H MRS, *in vivo* ^31^P MRS offers a more complete window on cell membrane metabolism. It can detect the individual PMEs and PDEs, but also the phosphorus species involved in energy metabolism, e.g. adenosine triphosphate (ATP), inorganic phosphate (Pi) and phosphocreatine (PCr). It has been shown that the PC/GPC ratio increases with increasing aggressiveness of breast cancer cells,^2^ and such a ratio can only be measured *in vivo* with ^31^P MRS and not with ^1^H MRS. Likewise, the chemotherapy treatment response is often accompanied by decreasing PMEs,^1^, ^20^, ^21^, ^22^ which can only be measured with ^31^P MRS. The decrease in tChol on response, which can be measured with ^1^H MRS, may be less specific.^10^ However, the intrinsic sensitivity of ^1^H MRS is much higher than that of ^31^P MRS.

Recent advances in ^31^P MRS, as reviewed by Khlebnikov et al,^23^ have resulted in an increased sensitivity. Moreover, the general formula of the sensitivity ratio between ^1^H and ^31^P, as known from the NMR field (γ^1^H/γ^31^P)^3^,^24^ is not what is practically encountered when applied in the human breast *in vivo*. First, in the regime of tissue load dominance of the receive coil, i.e. if noise from the sample dominates thermal noise from the coil, which is the case in human *in vivo* applications, the sensitivity ratio is reduced by approximately γ^1^H/γ^31^P to (γ^1^H/γ^31^P)^2^, as the thermal noise of a conducting sample is proportional to the resonance frequency.^24^ Second, when in the regime in which *T*_2_* substantially dominates over *T*_2_ (i.e. as a result of susceptibility differences between lipids and glandular tissue in the breast: *T*_2_* < < *T*_2_), the sensitivity ratio between ^1^H and ^31^P MRS is reduced by approximately the square root of the ratio γ^1^H/γ^31^P to (γ^1^H/γ^31^P)^3/2^. This includes two counteracting effects. The sensitivity of ^31^P is increased by γ^1^H/γ^31^P, because of a sharper line width (peak height) for ^31^P, as a result of the lower resonance frequency of the ^31^P nucleus relative to the ^1^H nucleus, but decreased by the square root of γ^1^H/γ^31^P, because of the longer sampling time of this sharper peak. (If *T*_2_* < < *T*_2_, then *T*_2_*(1H)/*T*_2_*(25P) ≈ (γ^1^H/γ^31^P)^−1^. The effect of the longer sampling time for ^31^P is analogous to decreasing the number of sampling averages, and varies with the reciprocal square root (γ^1^H/γ^31^P)^–1/2^.^24^ It should be noted that all of these sensitivity comparisons only hold under the assumption of RF tissue load dominance and susceptibility dominance.) Moreover, as ^31^P signals cannot be detected from adipose lipids, voxel selection in ^31^P MRS becomes substantially less critical. Third, when using multi‐echo sequences, such as AMESING (adiabatic multi‐echo spectroscopic imaging), the sensitivity of ^31^P MRS can be increased.^26^ Finally, polarization transfer (not used here) can potentially increase the sensitivity by a factor γ^1^H/γ^31^P.^27^, ^28^, ^29^, ^30^

In this study, we compare state‐of‐the‐art ^1^H MRS, incorporating all of the techniques mentioned, with AMESING ^31^P MRS to assess the reproducibility of detection of choline levels in the human breast in a group of eight healthy volunteers. Finally, we combine ^1^H and ^31^P MRS data to make an estimation of the concentrations of PMEs and PDEs in healthy fibroglandular tissue.

## 2 METHODS

### 2.1 Experimental details

A group of eight healthy female volunteers (21–30 years) underwent two MRI scan sessions of the right breast with a dual‐tuned ^1^H–^31^P quadrature coil (MR Coils BV, Drunen, the Netherlands)^21^ on a 7‐T scanner (Philips Healthcare, Cleveland, OH, USA). Each volunteer was scanned twice on the same day to minimize any effects of hormonal fluctuations/physiological variations on the measurements. The protocol consisted of imaging (scout, fat‐suppressed *T*_1_‐weighted imaging), a *B*_0_ map used for shimming, ^31^P magnetic resonance spectroscopic imaging (MRSI) and metabolite‐cycled ^1^H semi‐LASER spectroscopy. ^1^H spectroscopy was focused on the measurement of the tChol signal and was performed with a water‐ and fat‐suppressed semi‐LASER proton sequence^15^ with metabolite cycling^16^, ^17^ of the choline resonance at 3.2 ppm and a voxel of 15 × 15 × 15 mm^3^ carefully placed in fibroglandular tissue. The semi‐LASER sequence was performed with offset independent trapezoid pulses with a duration of 3.92 ms at a maximum *γB*_1_ of 1065 Hz and had a 95% inversion bandwidth of 6 kHz. During the first scan session, a screen print was made of the voxel placement (in three directions) and, during the second scan session, this screen print was used as a map to visually place the voxel at approximately the same position for the second scan session. As water was used as an internal reference for quantification, any variation in fibroglandular tissue content in the voxel would be accounted for and would not lead to different tChol concentrations as long as the tChol concentration is homogeneous over the fibroglandular tissue. Water suppression was performed by a VAPOR scheme^13^ and fat suppression by a MEGA scheme^14^ with narrow‐band adiabatic full passage (AFP) pulses. Metabolite cycling for tChol was performed by measuring a dynamic series of 2 × 14 scans for each TE (118, 119, 120 and 121 ms) and selectively inverting (with a narrow‐band AFP pulse with a frequency sweep of 300 Hz) the signal at 3.2 ppm in the even scans, and subsequently subtracting even from odd scans after phasing the residual water signal, which was a few per cent of the unsuppressed water signal. ^1^H spectroscopy was cardiac triggered with a TR of three heart beats for the choline scan (at four different TEs: 118, 119, 120 and 121 ms) and nine heart beats for the water reference scan with TE = 26 ms. The total scan time for the ^1^H spectroscopy protocol at an average heart rate of 60 beats/min was 6 min and 12 s.

^31^P MRSI was performed by AMESING^26^ with the following parameters: TR = 6 s; TE = 45 ms; matrix, 8 × 8 × 8; 4 × 2 × 4 cm^3^ (RL × AP × FH) voxels; spherical *k*‐space sampling; acquiring one free induction decay (FID) and five full echoes; a bandwidth of 8200 Hz and 256 data points for the echoes. The adiabatic pulses were driven with *γB*_1_ = 1700 Hz, and the pulse durations of the adiabatic half passage (AHP) and BIR‐4 refocusing pulses were 2 and 8 ms, respectively. Their respective 95% excitation/refocusing bandwidths were 1400 Hz and 1100 Hz. The total scan time of the ^31^P spectroscopy protocol was 25 min and 36 s. No proton decoupling was applied, as this contributes merely to SAR and not to the signal‐to‐noise ratio (SNR) *in vivo* in the breast at 7 T, because of the spectral line widths, which are several times the coupling constants between ^1^H and ^31^P. All volunteers gave written informed consent to participate in this study.

### 2.2 Post‐processing of ^31^P MRSI data and ^1^H semi‐LASER data

^31^P MRSI data were spatially Hanning filtered and subsequently apodized by 10 Hz with a Lorentzian line shape and zero filled to 2048 data points in the time domain. After zero‐ and first‐order phasing, the baselines of the FID spectra were corrected by fitting a second‐order spline. All spectral FIDs and echoes were frequency aligned to Pi. The voxel with the highest SNR was chosen for further analysis by maximizing the amount of glandular tissue in the selected voxel by voxel shifting.

^1^H spectra at the four different TEs were phased and frequency aligned on the residual water signal, and averaged subsequently. The averaged spectra from the even scans were subtracted from the averaged odd scans in order to obtain the tChol signal.

### 2.3 Spectral fitting of ^31^P data

The acquired data were analyzed on a group level and on an individual level. Spectral fitting was performed with Lorentzian line shapes in JMRUI^31^ using the AMARES^25^ algorithm, employing a priori constraints for the chemical shift difference between PE and PC of 0.5 ppm^32^ and 0.56 ppm for the chemical shift difference between (diacyl‐)glycerophosphatidylethanolamine (GPtE) + GPC and (diacyl‐)glycerophosphatidylcholine (GPtC).^33^ In addition, a 10‐Hz additional line width in the FID spectra for the PDEs, relative to Pi and PMEs, was set to compensate for the much shorter *T*_2_ times of the mobile phospholipid PDE signals in the breast.^22^, ^34^ Fibroglandular tissue also contains small amounts of adenosine monophosphate (AMP),^35^ with a chemical shift in between PE and PC at physiological pH.^36^ The signal of GPE in the breast overlaps partially with the GPtE‐MPL (membrane phospholipid) resonance, and concentrations of GPE in the breast are too low to give sufficient signal intensity for quantification. Line widths for PE, PC and Pi were kept free but identical, and line widths for γ‐nucleoside triphosphate (γ‐NTP) and α‐NTP were also kept free but identical. As the spectral resolution in the breast at 7 T is insufficient to distinguish multiplets from *J* coupling with protons, multiplets were not considered and fitted as single resonances. Spectral fitting with these a priori constraints was used to obtain the values of the line widths and chemical shifts for all metabolites per volunteer.

### 2.4 Spectral fitting of ^1^H data and calculation of tChol concentrations

Spectral fitting was performed with Lorentzian line shapes in JMRUI^31^ using the AMARES^25^ algorithm. The frequency of the singlet peak from tChol was constrained between 3.1 and 3.3 ppm and the line width was not constrained. In a second analysis, we verified the influence of constraint of the tChol line width to the line width of the water peak in the sum of even and odd spectra (i.e. the summed spectrum in which tChol should cancel out and residual water and fat peaks should not).

The quantification of tChol was based on a comparison with the reference water scan of the same voxel volume. Longitudinal and transverse relaxation times of water at 7 T were taken from Haddadin et al,^9^ and were 2.27 s and 36 ms, respectively. For tChol, the *T*_1_ value was taken to be equal to that of water and a *T*_2_ value of 200 ms was assumed (see Discussion).

### 2.5 *T*_2_ analyses of ^31^P metabolites

Metabolite *T*_2_ values were determined based on the weighted averaged group spectra (one FID and five echoes) of the eight volunteers. Each volunteer was measured twice, and so the group‐averaged FID is the Pi‐weighted average of 16 FID spectra. Weighted averaging was chosen to maximize SNR of the group spectra. Likewise, the group‐averaged echo spectra were also weighted with the Pi intensity of the FID. Weighted averaged spectra were subsequently spectrally fitted for PE, PC, Pi, GPC + GPtE, GPtC, PCr, γ‐NTP, α‐NTP and NAD in JMRUI^31^ with the AMARES^25^ algorithm. Spectral peak areas of all metabolites in the FID and echoes were fitted mono‐exponentially as a function of TE. The spectral amplitudes of PE, PC and GPC + GPtE were also fitted bi‐exponentially. For the PMEs, it was assumed that, at short TE, the signal shows contributions from nucleoside monophosphate, for which a *T*_2_ similar to that of γ‐NTP was chosen. The GPC + GPtE resonance was fitted assuming a *T*_2_ of GPtE similar to GPtC.

### 2.6 Reproducibility

All volunteers were measured twice on the same day (to minimize physiological influences) and the spectral fitting of these two datasets for each volunteer was used to calculate a standard deviation and a coefficient of variation for each metabolite in all volunteers. The standard deviation of the fitted relative peak area (with respect to the total ^31^P signal) *A*_*m,n*_ of a metabolite *m* in volunteer *n* can be written as:

(1) $${\mathrm{sd}}_{A_{m,n}}=\sqrt{\frac{{\left(\left\langle {\bar{A}}_{m,n}\right\rangle -A_{m,n,1}\right)}^{2}+{\left(\left\langle {\bar{A}}_{m,n}\right\rangle -A_{m,n,2}\right)}^{2}}{2}}$$

where $\left\langle {\bar{A}}_{m,n}\right\rangle$is the average of the fitted peak area of metabolite *m* over the two measurements of volunteer *n*. Subsequently, the coefficient of variation CoV in the fitted peak area of a metabolite *m* in volunteer *n* can be written as:

(2) $${\mathrm{CoV}}_{A_{m,n}}=100\cdot \frac{{\mathrm{sd}}_{A_{m,n}}}{\left\langle {\bar{A}}_{m,n}\right\rangle }$$

Analogously, we can define the average standard deviation and the average coefficient of variation for the peak area of a metabolite *m* over the whole group of eight volunteers as:

(3) $$\left\langle {\bar{\mathrm{sd}}}_{A_{m}}\right\rangle =\sqrt{\frac{\sum_{n=1}^{n=8}{\mathrm{sd}}_{A_{m,n}}^{2}}{8}}$$

and

(4) $$\left\langle {\bar{\mathrm{CoV}}}_{A_{m}}\right\rangle =100\cdot \frac{\left\langle {\bar{\mathrm{sd}}}_{A_{m}}\right\rangle }{\left\langle {\bar{A}}_{m}\right\rangle }$$

respectively.

## 3 RESULTS AND DISCUSSION

### 3.1 *T*_2_ analysis of ^31^P metabolites in fibroglandular breast tissue

**Figure 1:**
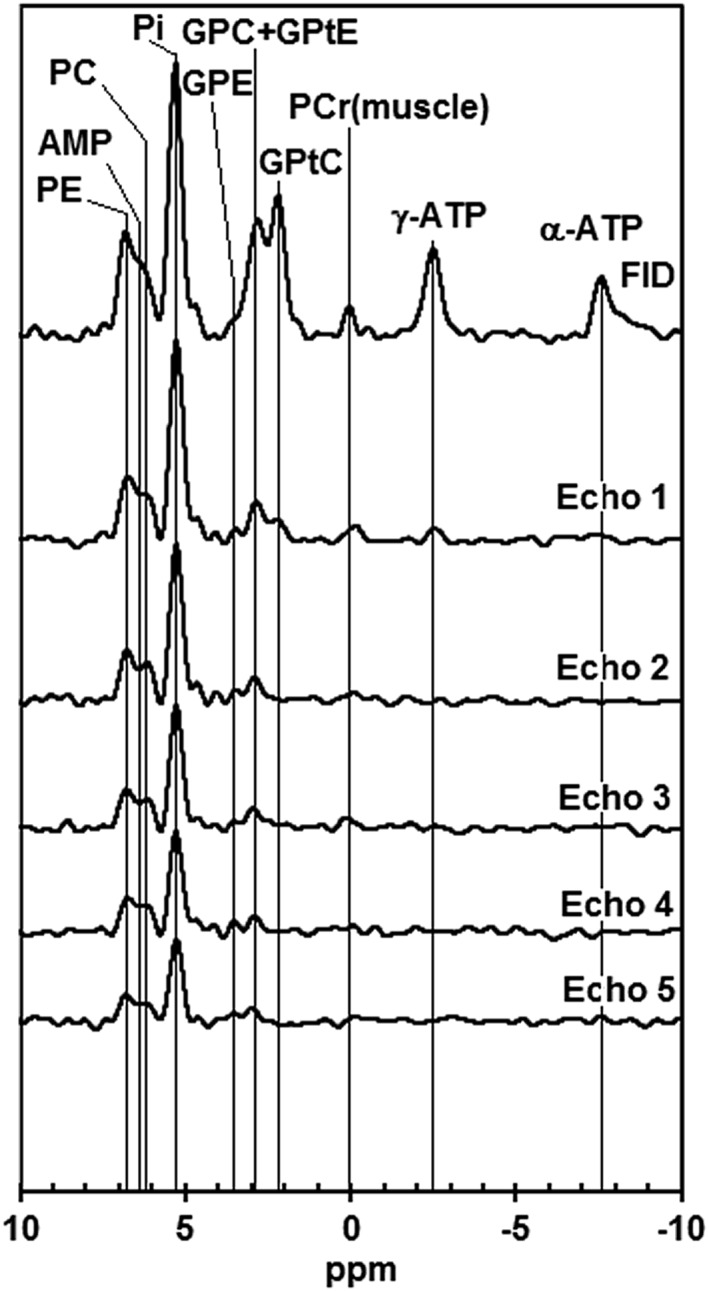
Averaged free induction decay (FID) and echo ^31^P magnetic resonance spectra (echo spacing, 45 ms) for a group of eight healthy volunteers scanned twice. AMP, adenosine monophosphate; ATP, adenosine triphosphate; GPC, glycerophosphocholine; GPE, glycerophosphoethanolamine; GPtC, (diacyl‐)glycerophosphatidylcholine; GPtE, (diacyl‐)glycerophosphatidylethanolamine; PC, phosphocholine; PCr, phosphocreatine; PE, phosphoethanolamine; Pi, inorganic phosphate

For ^31^P spectroscopy, the measured line widths are in the range 35–50 Hz (0.3–0.4 ppm). The group‐averaged FID and echo spectra (echo spacing, 45 ms) based on the spectra of the eight volunteers are shown in Figure 1. It should be noted that the peak labeled GPtC is no longer visible from Echo 2 onwards, comparable with the rapid disappearance of γ‐NTP. The short apparent *T*_2_ of γ‐NTP is affected by *J* modulation because of homonuclear ^31^P coupling.^37^ In addition, it should be noted that the peak labeled GPC + GPtE loses substantial intensity from FID to the first echo (GPtE has a short *T*_2_), after which the decrease in intensity is much slower, leaving only the slowly decaying GPC signal. The short *T*_2_ PDE signals (GPtC and GPtE) are thought to originate from highly mobile membrane phospholipids.^34^, ^38^, ^39^ In the FID spectrum, there appears to be some signal between the peaks labeled PE and PC that seems to disappear in the echoes; this signal could be nucleoside monophosphate (NMP).^35^

The β‐NTP signal could not be observed above noise level and therefore the spectra are only shown between 10 and −10 ppm. The reason for the apparent absence of the β‐NTP signal in the FID spectrum may be two‐fold. First, the 95% excitation bandwidth of the AHP pulse is only 1400 Hz and the transmitter offset is approximately 2500 Hz away from the β‐NTP resonance. Second, the β‐NTP resonance is dependent on the Mg^2^^+^ concentration, which may be low in healthy breast fibroglandular tissue.

The group‐averaged spectra depicted in Figure 1 allow for a pH estimation of fibroglandular tissue based on the chemical shift of Pi. As the small PCr signal most probably originates from voxel bleeding from the pectoralis muscle (which may have a *B*_0_ offset because shimming was aimed at the glandular tissue), it is safer to use the signal from GPC and/or α‐NTP as chemical shift reference value and take, for these resonances, chemical shift values that have been measured *in vivo*, in the presence of PCr, which is by definition 0.0. Here, we adopt for GPC and α‐NTP, values of 2.97 ppm and −7.57 ppm, respectively, as measured in mammalian brain.^40^ For the FID spectrum, we use the α‐NTP resonance as it is most clear, whereas the GPC signal in FID is affected by mobile phospholipid; for the echo spectra, we take GPC as chemical shift reference. In this way, we have six measurements (one FID and five echoes) for the chemical shift of Pi which is, on average, 5.31 ± 0.05 ppm, corresponding to a pH value of 7.51 ± 0.07.

**Figure 2:**
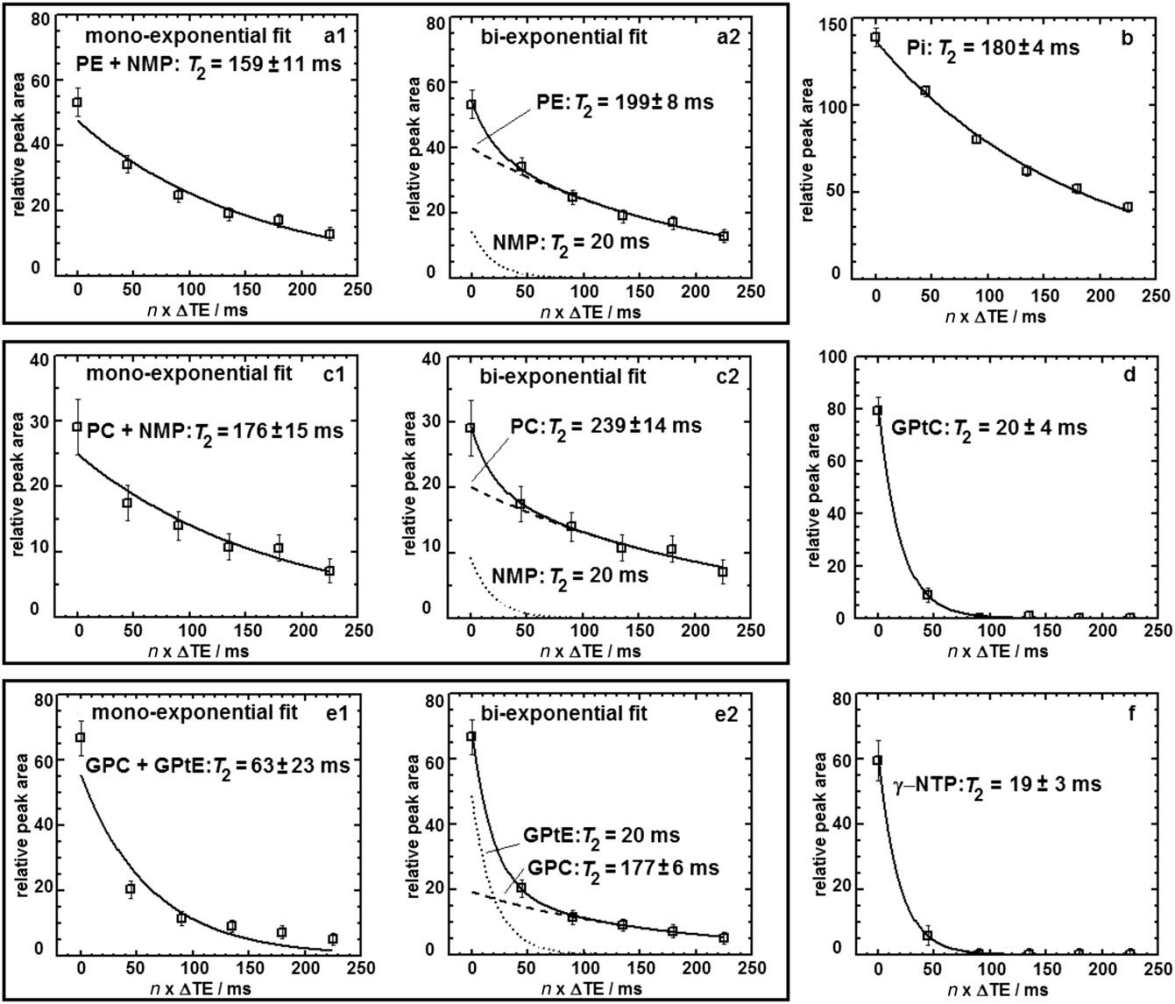
Transverse relaxation times *T*_2_ fitted for various ^31^P metabolites in fibroglandular tissue, derived from the weighted average group spectra [one free induction decay (FID) and five echoes] of eight healthy volunteers measured twice. (a1) Mono‐exponential *T*_2_ fit for the combined resonance PE + NMP. (a2) Bi‐exponential *T*_2_ fit for the combined resonance PE + NMP. (b) (Mono‐exponential) *T*_2_ fit for Pi. (c1) Mono‐exponential *T*_2_ fit for the combined resonance PC + NMP. (c2) Bi‐exponential *T*_2_ fit for the combined resonance PC + NMP. (d) (Mono‐exponential) *T*_2_ fit for GPtC. (e1) Mono‐exponential *T*_2_ fit for the combined resonance GPC + GPtE. (e2) Bi‐exponential *T*_2_ fit for the combined resonance GPC + GPtE. (f) (Mono‐exponential) *T*_2_ fit for γ‐NTP. GPC, glycerophosphocholine; GPtC, (diacyl‐)glycerophosphatidylcholine; GPtE, (diacyl‐)glycerophosphatidylethanolamine; NMP, nucleoside monophosphate; NTP, nucleoside triphosphate; PC, phosphocholine; PE, phosphoethanolamine; Pi, inorganic phosphate

Figure 2 shows the *T*_2_ fits for the different ^31^P metabolites. It should be noted that the FID signal amplitudes of PE + NMP, PC + NMP and GPC + GPtC all appear well above the fitted mono‐exponential curve. The additional bi‐exponential fit for these resonances shows a better agreement with the data. As the *T*_2_ values of the NMP and GPtC components are not known, we assume a similar *T*_2_ for NMP as for the apparent *T*_2_ of γ‐NTP that we measured and, likewise, a similar *T*_2_ of GPtC as for the GPtE component that we measured. An overview of the fitted T2 data is shown in Table 1.

**Table 1:** Fitted *T*_2_ values of ^31^P metabolites in healthy fibroglandular breast tissue

Metabolite	*T*_2_ (ms)	Metabolite	*T*_2_ (ms)
PE*	199 ± 8	GPC*	177 ± 6
PC*	239 ± 14	GPtC	20 ± 4
Pi	180 ± 4	γ‐NTP	19 ± 3

* Fitted bi‐exponentially with an additional short *T*_2_ compound of 20 ms (NMP for PE and PC; GPtE for GPC).

GPC, glycerophosphocholine; GPtC, (diacyl‐)glycerophosphatidylcholine; GPtE, (diacyl‐)glycerophosphatidylethanolamine; NMP, nucleoside monophosphate; NTP, nucleoside triphosphate; PC, phosphocholine; PE, phosphoethanolamine; Pi, inorganic phosphate.

The relative metabolite abundances expressed as a percentage of the total ^31^P signal, for this group of volunteers measured twice, as obtained from the data in Figures 1 and 2, are: PE = 8%; AMP = 5%; PC = 4%; Pi =29%; GPE ~ 0%; GPC = 4%; GPtE =10%; GPtC =17%; γ‐NTP = 13%; α‐NTP = 8%; NADP =2%.

### 3.2 Reproducibility of ^31^P measurements

**Figure 3:**
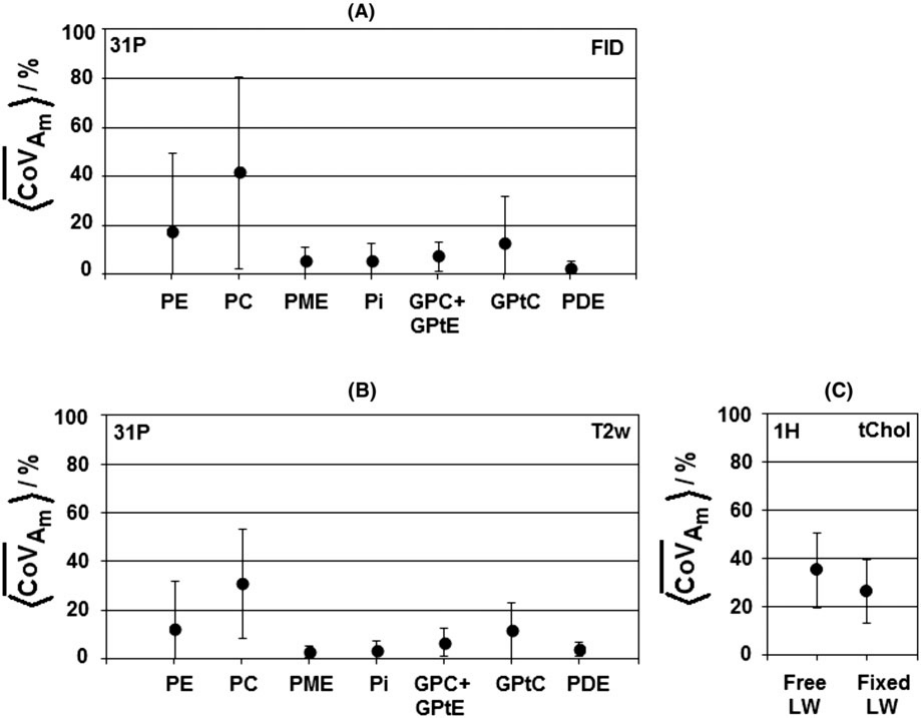
Coefficient of variation in metabolite peak amplitudes (Equation (4)), as fitted with JMRUI. A, ^31^P magnetic resonance spectroscopy (MRS) derived from free induction decay (FID) data. B, ^31^P MRS derived from *T*_2_‐weighted (T2w) data. C, ^1^H MRS total choline (tChol), with free line width (LW) and fixed LW for tChol. GPC, glycerophosphocholine; GPtC, (diacyl‐)glycerophosphatidylcholine; GPtE, (diacyl‐)glycerophosphatidylethanolamine; PC, phosphocholine; PDE, phosphodiester PE, phosphoethanolamine; Pi, inorganic phosphate; PME, phosphomonoester

The average relative errors (Equation (4)) in the amplitudes of the different metabolite signals, derived from spectral fitting of the FID spectra and the *T*_2_‐weighted (T2w) average spectra, are shown in Figure 3a,b. Errors on the individual monoester signals are largest because these signals have, on average (see Figure 1), the lowest SNR, overlap partially and are also influenced by the presence of a disturbing signal from NMP. The sum of monoesters, however, shows only small variability. Note the reduction in the relative error and standard deviation of the relative error when making use of T2w spectra, which combine FID and echo spectra, relative to FID spectra, which results in increased SNR and more reliable fitting.

### 3.3 ^1^H spectroscopy

**Figure 4:**
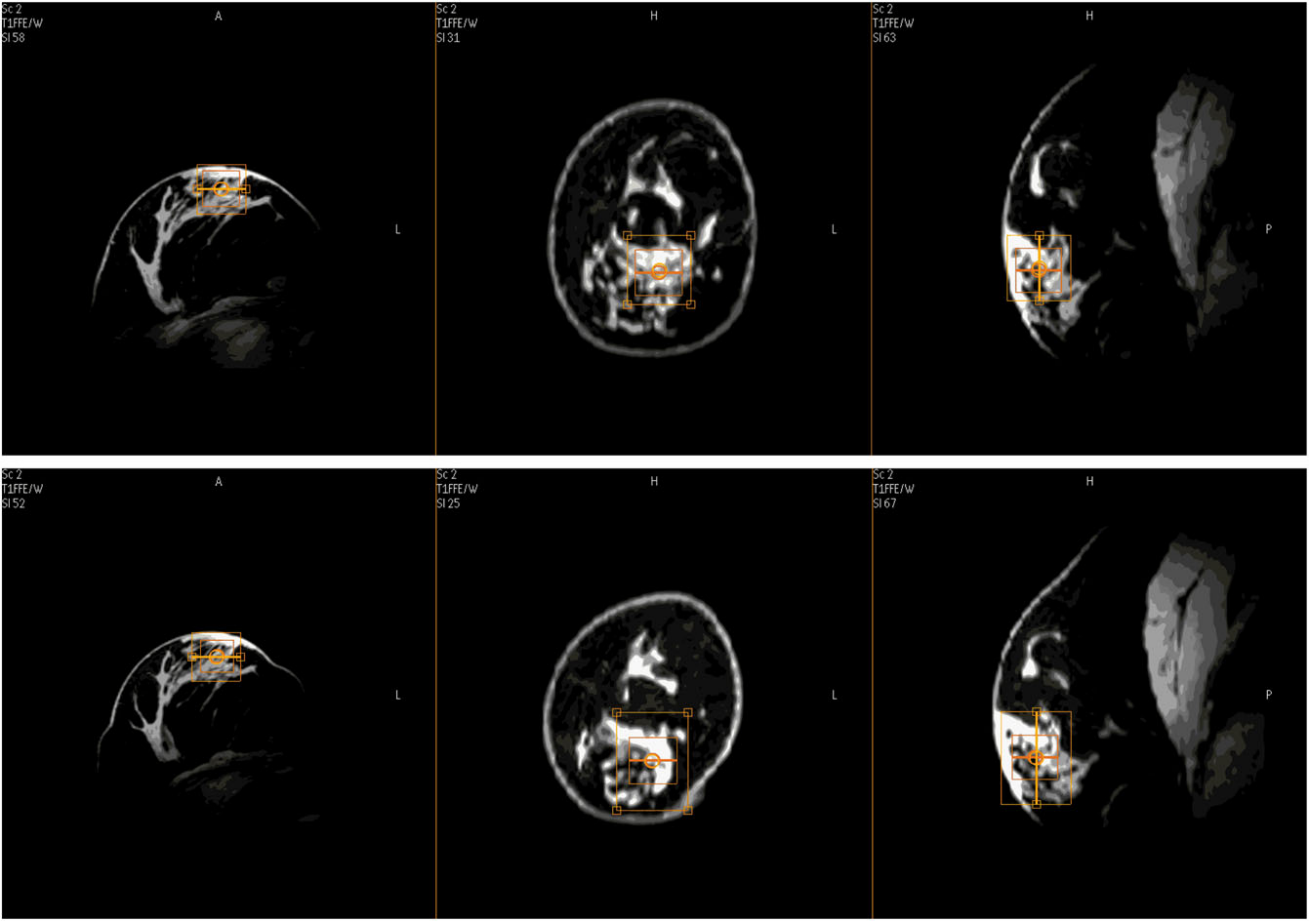
Example of voxel placement in three different planes of view for duplicate ^1^H semi‐LASER magnetic resonance spectroscopy in a volunteer, the inner square is the selected voxel. Top row, first measurement series. Bottom row, voxel placement for second measurement series

Figure 4 shows an example of the voxel placement for the semi‐LASER ^1^H MRS in a single volunteer for the duplicate measurements. The line width of water in the small ^1^H MRS voxel is in the range 20–50 Hz (0.07–0.17 ppm). The tChol concentrations, determined in duplicate, for a voxel of fibroglandular tissue of the eight volunteers are shown in Table 2. In the case of volunteers 3 and 5, one of the two determinations gave insufficient signal to determine a tChol concentration. Spectral fitting was performed with an unconstrained line width for tChol and also with a fixed line width for tChol, equal to the line width of the water signal, whilst adding the (frequency aligned) odd and even scans (i.e. tChol cancels out and residual water and fat peaks do not). The group‐averaged tChol concentration of the data analyzed with a free line width for tChol, as well as with a fixed line width, from Table 2 is 0.54 mM (or, more precisely, mmol/kg_water_). Correcting for the water content of fibroglandular tissue being approximately 60%,^41^ this would reduce to 0.32 mM.

**Table 2:** Total choline (tChol) concentration of fibroglandular tissue of a group of eight healthy volunteers. Data are calculated as choline, i.e. assuming that all signal between 3.1 and 3.3 ppm is choline based (nine protons). *T*_1_ and *T*_2_ values for water were taken from Haddadin et al.^9^ Errors are based on Cramer–Rao lower bounds from JMRUI. Analyses were performed with a free line width for tChol and with a fixed line width equal to the line width of the water signal

Volunteer	Measurement 1 tChol (mM)		Measurement 2 tChol (mM)	
	Free line width	Fixed line width	Free line width	Fixed line width
1	0.4 ± 0.1	0.7 ± 0.1	0.4 ± 0.1	0.5 ± 0.1
2	1.0 ± 0.3	1.1 ± 0.3	1.1 ± 0.3	1.0 ± 0.3
3	(0)	(0)	0.6 ± 0.1	0.5 ± 0.1
4	0.5 ± 0.1	0.6 ± 0.1	1.0 ± 0.2	0.6 ± 0.1
5	0.7 ± 0.2	0.5 ± 0.2	(0)	(0)
6	0.6 ± 0.1	0.5 ± 0.1	0.6 ± 0.1	0.5 ± 0.1
7	0.6 ± 0.2	0.6 ± 0.2	0.3 ± 0.1	0.6 ± 0.2
8	0.4 ± 0.1	0.4 ± 0.1	0.4 ± 0.1	0.3 ± 0.1

The calculated tChol concentration depends on the *T*_1_ and *T*_2_ values that were assumed for tChol. As these values are not known in the breast at 7 T, they had to be estimated. For the *T*_1_ of tChol, we assumed a value similar to the *T*_1_ of water (2.27 s) at 7 T, as tabulated by Haddadin et al.^9^ For the *T*_2_ of tChol, we assumed a value of 200 ms. Reported *T*_2_ values of tChol in the breast at 1.5 T range between 181 and 360 ms, whereas, at 4 T, a value of 399 ± 133 ms was measured, as reviewed by Haddadin et al.^9^ A value of 178 ± 28 ms for tChol was measured at 9.4 T in rat brain.^42^ Therefore, a value in the range 150–250 ms for *T*_2_ of tChol at 7 T seems plausible. A value of *T*_2_ = 150 ms for tChol would lead to approximately 30% higher tChol concentrations than at *T*_2_ = 200 ms, and a value of 250 ms would lead to approximately 20% lower concentrations than reported here.

The relative error in the ^1^H tChol determination, calculated analogously to the ^31^P data (Equation (4)), as shown in Figure 3c, is 34% for the spectral fitting with free line width and 26% for the spectral fitting with fixed line width for tChol.

The interpretation of the relative errors depicted in Figure 3 should also take into consideration the difference in voxel sizes used for ^31^P and ^1^H spectroscopy. Here, we used 4 × 2 × 4 cm^3^ (nominal resolution) voxels for ^31^P MRSI of healthy glandular tissue, whereas, in a clinical setting with breast cancer patients, we would use smaller voxels of 2 × 2 × 2 cm^3^. Considering the acquisition‐weighted *k*‐space sampling and the applied Hanning filter in the MRSI sequence, the true voxel size in our study is even larger: an ellipsoid of approximately 82 cm^3^. In a worst case, this would mean an increase in the coefficient of variation for ^31^P spectroscopy of a factor of four on using the 2 × 2 × 2 cm^3^ nominal voxel size. As the larger voxel size does not imply that these voxels are completely filled with glandular tissue, they may contain fatty tissue (without ^31^P signal) as well, such that the factor of four in the coefficient of variation is the limiting case.

Although the intrinsic sensitivity of ^31^P MRS remains less than that of ^1^H MRS, the absence of disturbing signals from lipids and water and the insensitivity of the AMESING sequence to *B*_0_ inhomogeneities provide substantial advantages of ^31^P MRS over ^1^H MRS in the robust detection of phospholipid metabolites. With the larger voxel sizes, longer scan times and absence of artifacts, ^31^P MRS could detect the phospholipid metabolite signals with an order of magnitude improved coefficient of variation (Figure 3) than with ^1^H MRS.

**Figure 5:**
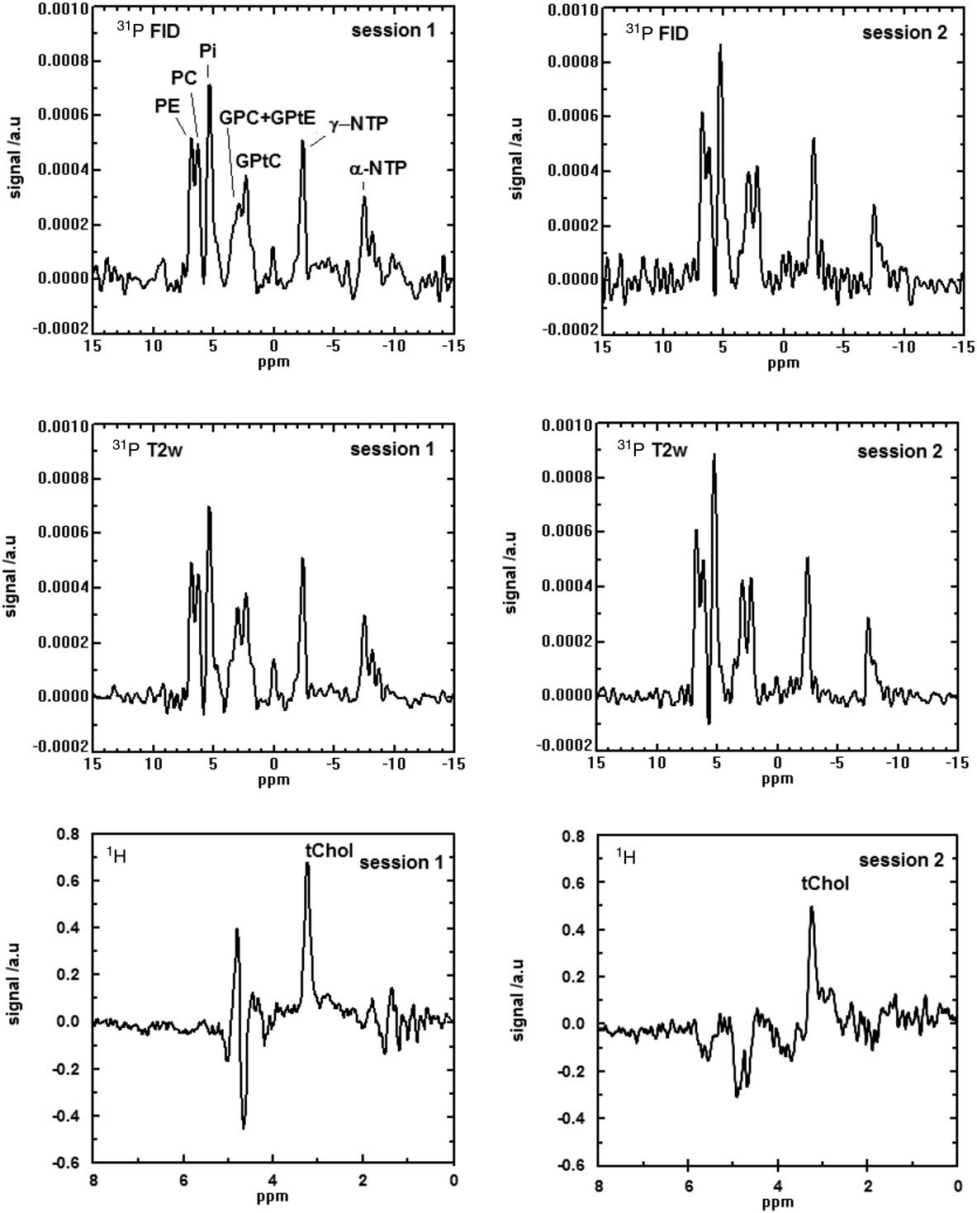
Duplicate ^31^P spectra [free induction decay (FID) and *T*_2_‐weighted] and ^1^H total choline (tChol) spectra for volunteer 2. GPC, glycerophosphocholine; GPtC, (diacyl‐)glycerophosphatidylcholine; GPtE, (diacyl‐)glycerophosphatidylethanolamine; NTP, nucleoside triphosphate; PC, phosphocholine; PE, phosphoethanolamine; Pi, inorganic phosphate

Figure 5 shows an example of ^31^P MR spectra and metabolite cycled ^1^H MR tChol spectra for volunteer 2 measured in duplicate. Although the noise level is expected to be the same in all ^31^P acquisitions, the noise pattern of the ^31^P spectrum in the top right of Figure 5 seems to include a higher spectral noise density than in the remaining spectra. This may be caused by truncation artifacts as a result of the relatively short acquisition window of 16 ms of FID acquisition, which affects the spectral region most strongly around sharp and high‐intensity peaks.

Estimates of the average PME and PDE concentrations in healthy fibroglandular tissue based on a combination of ^31^P and ^1^H MRS, using the ^31^P data shown in Figures 1 and 2, and the average tChol value of Table 1 of 0.54 mM, are PE = 0.4 mM (two protons), PC = 0.2 mM (nine protons) and GPC = 0.2 mM (nine protons), where it was assumed that the free choline concentration can be neglected. The estimated concentrations of PMEs and PDEs are therefore an upper limit. Correction for 60% water content of fibroglandular tissue^39^ would lead to approximate values, expressed in mmol/kg of tissue (assuming a tissue density of approximately 1 kg/L), which are 60% of the values expressed in mM. An assessment by liquid chromatography‐mass spectrometry of three samples of healthy breast tissue by Mimmi et al^43^ led to values for PC in the range 0.03–0.21 mmol/kg; this corresponds well with our value for PC expressed in the same units (mmol/kg of tissue) of 0.1 mmol/kg_tissue_.

This study shows the feasibility of combining ^31^P MRSI with localized ^1^H MRS *in vivo* in the breast at 7 T. The combination of these techniques may be of value in monitoring the response to chemotherapy in breast cancer patients, where it may possibly better discriminate between responders and non‐responders than ^31^P or ^1^H MRS alone. From *ex vivo* analysis^43^, ^44^ of breast cancer samples, it is known that the free choline concentrations are small, compared with PC; therefore, combined ^31^P and ^1^H MRS *in vivo* in breast cancer will enable, to a good approximation, absolute concentration measurements of key metabolites of cell membrane metabolism.

## 4 CONCLUSIONS

*In vivo* ^31^P MRS offers a better window on cell membrane metabolism and energy metabolism than does tChol derived from ^1^H MRS. The ^31^P MRS reproducibility of the individual PMEs was similar to that of ^1^H tChol MRS. The reproducibility of Pi, the sum of PMEs and the sum of PDEs with ^31^P MRSI was superior to the reproducibility of ^1^H MRS for tChol. Upper limits for the PME and PDE concentrations in healthy fibroglandular breast tissue derived from this study are [PC] = 0.1 mmol/kg, [GPC] = 0.1 mmol/kg and [PE] = 0.2 mmol/kg.
